# Suicide in Married Women: An Autopsy Study

**DOI:** 10.7759/cureus.41510

**Published:** 2023-07-07

**Authors:** Shashmira B Tonse, Swati Sonawane, Haris V R

**Affiliations:** 1 Forensic Medicine & Toxicology, Dr. D. Y. Patil Medical College, Nerul, Navi Mumbai, IND

**Keywords:** employment, education, domestic abuse, dowry, married indian women, suicide

## Abstract

To consider death by suicide, as a method to escape their problems, is accepting defeat. Before embarking on the journey of marriage, one envisions the best of life and has a lot of hope for their future life. However, demands of dowry and domestic abuse by the husband can cut such visions short. Suicidal deaths among women especially married women have been increasing in Indian society. Various cultural, religious, and social values have a major role to play. In our study, we analyzed suicidal deaths in married women and tried to find the socio-demographic findings that have led these women to commit suicide. The autopsies were conducted from January 2014 to July 2015 at Kempegowda Institute of Medical Sciences, Bangalore. The highest incidence of suicide was found in the age group of 26-32 years, who were homemakers and within seven years of marriage. In a maximum of cases, abuse for dowry or other reasons was quoted as the reason to commit suicide. We also found that most of the decedents choose to hang themselves to commit suicide followed by the consumption of poison.

## Introduction

Suicidal death is a violent death where the decedent deliberately and successfully kills oneself. The World Health Organisation (WHO) estimates that around 800,000 suicide deaths occur worldwide every year, with an annual global age-standardized suicide rate of 10.5 per 100,000 population [1]. India accounts for 36.6% of global suicide deaths in women [2]. According to the latest accidental deaths and suicides in India, the Annual Report of 129 females commit suicides on a daily basis out of which 60 are housewives in India. Karnataka has contributed to 8% of suicides in India, whereas Bangalore City has reported higher numbers of suicides [3].

Suicidal deaths of married women have been an increasing trend in Indian society in recent years. Various cultural, religious, and social values play an important role in this regard. The patriarchal structure in India downplays the value of a woman in society. Despite the strides the country is making in terms of a woman’s health, her rights to education, and equal opportunity, the birth of a female child is still frowned upon. Unless a woman is under the shadow of the care of a man she is considered incomplete. Being a widow or a divorcee is considered a social death sentence at all levels of society in India.

Married men and women have a lower suicide risk than unmarried, divorced, or widowed in Western countries [4,5]. However, Marriage in India doesn’t afford women the same protection from suicide. In India, harassment for dowry or domestic abuse by their husband or in-laws may push a woman towards committing such an act.

Dowry in the Indian context, refers to any moveable or immovable property that is gifted to the groom on demand, by the bride's parents, which is diametrically opposed to the concept of Mahr or Mehr amongst the Islamic community. Culturally, it is an accepted practice to exchange expensive gifts at the time of marriage between the two parties. But, if the nature and cost of these gifts fail to satisfy the groom and his family, demands are put forth. If such demands are beyond the ability of the bride’s family to fulfill, these expectations can take a heavy toll. The demand for dowry is prohibited by the Dowry Prohibition Act of 1961 and is punishable by law. In many instances, such demands are not reported, as it could decrease the prestige of the bride and her family. In situations where the dowry demands are not met, after marriage the bride is threatened by physical abuse or is emotionally tortured and harassed to convince her family to fulfill the demands of the groom or his parents.

Such abuse and lack of financial independence can strain a woman’s psyche, making her unable to put herself above the needs of her family. Over time such discriminations erode the confidence and resilience of women making them prone to mental disabilities like depression which in itself is a leading cause of suicide in developed countries.

## Materials and methods

The present study was conducted from January 2014 to July 2015 in the Department of Forensic Medicine and Toxicology at Kempegowda Institute of Medical Sciences (KIMS), Bangalore, Karnataka State, India, a tertiary center. Using a pre-tested structured schedule, all cases of suicidal and suspected suicidal deaths in females in the age group 18 to 65 years brought to the KIMS Hospital Mortuary, Bangalore, for autopsy were included in the study. Cases of suicide in which the body was decomposed, unknown, or unclaimed, and those cases where the manner of death was initially thought to be suicidal but later proved to be homicidal or accidental were excluded. A total of 126 cases were included in the study out of which two cases were excluded as the bodies were in a state of advanced decomposition. Out of these, 91 cases were of women who were married and they were included in the present study.

Information regarding the decedent with regard to the familial situation was collected from the Inquest report that was submitted by the investigating officer of each case. In certain cases, where the decedent was admitted prior to death, in-patient records were accessed for information regarding the history as given by the decedent or the family members. The post-mortem report was studied for the cause of death and the method that was employed by the decedent. In cases, where the decedent had consumed poison to commit suicide, blood samples from the liver, kidney, stomach, and its contents were collected, sealed, and sent to the State Forensic Science Laboratory, Madiwala, Bengaluru while maintaining a chain of custody. The relevant samples/viscera were subjected to chemical analysis on autopsy to establish the poison consumed in suspected cases of poisoning.

A standardized proforma specially designed for this purpose was used and filled in each case after detailed interviews with the investigating officials and the relatives/friends of the deceased and hospital records etc., to gather information regarding the age, socio-economic background, level of education, occupation, marital status, any history of domestic abuse or dowry demands, and history of physical/ mental diseases and presence of any other stressors. The religion of the decedent was classified as Hindu, Muslim, Buddhist, Christian, Sikh, or others (Parsi, Jain, etc.). Marital status was classified as being married, widowed, divorced or separated, and never married. The duration of marriage before death was divided into less than seven years, seven to 10 years, 10 to 20 years, and more than 20 years of marriage. Educational status was classified based on literacy, where illiterate is a person who cannot read or write; primary education where one has completed her education upto the IV standard; middle school as those who have completed their education upto VII standard; secondary school as one who has completed their education upto X standard; Junior college as one who has completed XII standard, Graduate as one who has completed their graduate degree and Post-graduate as one who has completed their post-graduate degree from any university. Occupational status was classified as Homemaker who is one who is purely engaged in household duties but doing no other productive work to augment the family finances, Labourer/domestic help as one who works for wages in cash (on a daily or monthly basis), employed as one who is a salaried person in Government, semi-government or private organisations, Unemployed as one who has completed her education but is not currently employed and Student as a full-time students college or appearing for exams (multiple attempts at final senior secondary exams).

The socio-economic status of the victim was based on modified Kuppuswamy’s classification (Modified for 2014) (Table 1). Three characteristics were considered viz., Education, Occupation, and Family Income. The weighted score of each of these three characteristics was taken. Based on the total score the victim was assigned to appropriate social class.

**Table 1:** Modified Kuppuswamy’s classification of socio-economic status (Modified for 2014) Reference no. [6]

Education	Score
Professionals or honours	7
Graduate or postgraduate	6
Intermediate or post high school diploma	5
High school certificate	4
Middle school certificate	3
Primary school certificate or literate	2
Illiterate	1
Occupation	Score
Professional	10
Semi-professional	6
Clerk, shop owner, farmer	5
Skilled worker	4
Semi-skilled worker	3
Unskilled worker	2
Unemployed	1
Family income Modified for 2014	Score
≥36,997	12
18,498 - 36,996	10
13,874 - 18,497	6
9,249 - 13,873	4
5547 - 9248	3
1866 - 5546	2
≤1865	1
Total score (calculated based on the above factors)	Socio-economic class
26-29	Upper class
16-25	Upper middle class
11-15	Lower middle class
5-10	Upper lower class
<5	Lower class

Being a descriptive study, a purposive sampling technique was used. All data were manually entered into an Excel sheet and was analyzed statistically by presenting the data in the form of appropriate tables and graphs and computing the proportions and percentages. The protocol for the present study was presented before the Ethical Committee of Kempegowda Institute of Medical Sciences, Bangalore, and the actual study started after getting approval for the same.

## Results

In the above-mentioned study period, 951 medico-legal autopsies were conducted at Kempegowda Institute of Medical Sciences Mortuary, Bangalore. Out of the 951 cases autopsied, 400 were suicidal deaths, out of which 146 (36.5%) were females committing suicide and 254 (63.5%) were males. Out of the 146 suicidal deaths in females, 126 of the females committing suicide were in the age group 18-65 years and were included. Two cases were excluded as the bodies were decomposed. Amongst the rest of the 124 cases, 91 were married and included in the present study. Amongst women committing suicide 73.38% were married (Table 2).

**Table 2:** Socio-demographic profile of married females committing suicide.

Marital Status		Number		Percentage	
Married		79		86.8	
Divorced/Separated		7		7.69	
Widowed		5		5.49	
Duration of Marriage		Number		Percentage	
Less than 7 years		36		39.56	
7 – 10 years		23		25.27	
10 – 20 years		23		25.27	
More than 20 years		9		9.89	
Age group (years)		Number		Percentage	
18 – 25		21		23.07	
26 – 32		34		37.36	
33 – 39		16		17.58	
40 – 49		10		10.98	
50 – 65		9		9.89	
Literacy		Number		Percentage	
Illiterate		9		9.89	
Completed primary schooling		12	82	13.19	90.1%
Completed middle schooling		27		29.67	
Completed secondary schooling		10		10.99	
Completed junior college		6		6.59	
Completed Graduation		26		28.57	
Completed Post graduation		1		1.09	
Religion		Number		Percentage	
Hinduism		64		70.32	
Christianity		1		1.09	
Islam		5		5.49	
Occupation		Number of cases		Percentage	
Housewife		73		80.21	
Employed		10		10.98	
Labourer/domestic help		5		5.49	
Unemployed		3		3.29	
Socio-economic Status (SES)		Number of cases		Percentage	
Upper (I)		2		2.19	
Middle	Upper Middle (II)	31	73	34.07	80.22
	Lower Middle (III)	42		46.15	
Lower	Upper Lower (IV)	11	16	12.09	17.58
	Lower Lower (V)	5		5.49	
Total		91		100	

The above table shows that the total number of married females committing suicide was 91; amongst them, the women who were married at the time of committing suicide were 79 (86.8%) of the total cases, seven (7.69%) women were divorced or separated from their husbands and five (5.49%) women were widowed at the time of committing suicide. Among the victims, the maximum number was in the category of being married for less than seven years with 36 (39.56%) cases. This was followed by those married for 7-10 years and 10-20 years accounting for 23 (25.27%) cases each. The least number was in the category of being married for more than 20 years, 9 (9.89%). We found that the highest number of victims belonged to the age group of 26-32 years, accounting for 34 (37.36%) cases followed by the age group of 18-25 years (23.07%). The least number of cases was observed in the age group of 50-60 years accounting for 9 (9.89%) cases. When we compared the educational status of the victims it was found that, the highest number of victims were in the group that completed their middle schooling, i.e., 27 (29.67%) which was closely followed by victims who have completed their graduation which accounted for 26 (28.57%) of the cases. The least number of victims had completed their post-graduation, accounting for one (1.09%) case. In our study, illiterate women accounted for nine (9.89%) cases. Religious distribution showed that; most of the women followed Hinduism, accounting for 64 (70.32%) cases, followed by those following Islam and those following Christianity, accounting for one (1.11%) case and five (5.49%) cases respectively. Housewives comprised the largest group of victims accounting for 73 (80.21%) cases. Laborers or domestic help made up the least number of victims accounting for five (5.49%) cases. In this study it was found that the most vulnerable socio-economic status for suicidal deaths in women is the middle class, accounting for 73 (80.22%) cases, followed by the lower class which made up 16 (17.58%) cases.

Table 3 shows that the maximum number of decedents chose to commit suicide by hanging which accounted for 76 (83.51%) cases, which was followed by consumption of an unknown poison which accounted for eight (8.79%) cases. In the present study, it was found that four (4.35%) women chose to burn themselves to commit suicide and two (2.20%) chose to jump from a height. In one (1.09%) case the deceased chose to incise her throat and hang afterward.

**Table 3:** Distribution of victims based on the method used

Method	Number	Percentage
Hanging	76	83.51
Hanging + self-cut throat	1	1.09
Consumption of poisonous substance	8	8.79
Fall from height	2	2.19
Burns	4	4.39
Total	91	100

Table 4 shows that the preferred poison among married women in the study was Organophosphate pesticide which accounted for five (62.5%) cases. Aluminium phosphide pesticide and diazepam tablets were used in one (12.50%) case each. In one case (12.50%) the chemical analysis was negative.

**Table 4:** Distribution of poisoning victims based on the choice of poison consumed

Type of poison consumed		Number		Percentage	
Agricultural poisons	Organophosphates	6	5	50	62.50
	Aluminium Phosphide		1		12.50
Pharmaceutical drugs (Diazepam tablets)		1		12.50	
Not detected in the chemical analysis		1		12.50	
Total		08		100	

Table 5 shows that the most common cause for suicide in married females was physical and mental torture for dowry or domestic abuse which accounted for 21 (23.07%) cases, which was followed closely by anxiety as a result of ill-health which accounted for 18 (19.78%) cases. Psychiatric disease or depression was the precipitating factor for committing suicide in 14 (15.38%) cases. In nine (9.89%) cases, married women committed suicide due to financial problems. Suicide due to grief due to the ill health of a family member or due to the loss of her spouse or first-degree relative accounted for eight (8.79%) cases. In eight (8.79%) cases, the cause was undetermined. In seven (7.69%) cases and four (4.39%) cases married women committed suicide due to a trivial fight with the husband and failure of marriage respectively. Some form of sexual harassment leading to suicide was seen in two (2.19) cases.

**Table 5:** Distribution of victims based on the reason for committing suicide

Reason for committing suicide			Number		Percentage		
Physical and mental torture		For dowry	9	21	9.89	23.07	
		For other reasons	12		13.18		
Depression due to ill health		Gynaecological problems	12	18	13.18	19.78	
		Surgical diseases	1		1.09		
		Medical diseases	5		5.49		
Psychiatric diseases or depression			14		15.38		
Grief D/T	Ill health of a family member		2	8	2.19		8.79
	loss of spouse/ family member		6		6.59		
Trivial fight with family members/husband			7		7.69		
Sexual harassment			2		2.19		
Financial problems			9		9.89		
Love/Marriage failure			4		4.39		
Unknown reason			8		8.79		
Total			91		100		

## Discussion

In the study period, a total of 951 cases were autopsied, amongst which 146 (36.5%) were females committing suicide and 254 (63.5%) were males. Out of the 146 suicidal deaths in females, 126 of the females committing suicide were in the age group 18-65 years. Two cases were excluded as the bodies were decomposed. Amongst the rest of the 124 cases, 91 were married and included in the present study. Amongst women, married women constitute 73.38% in the present study, which is consistent with the findings of other authors [7-12]. Pradhan et al. [7], Dandona et al. [8], Karthik et al. [10], Prajapati et al. [11], and Khan et al. [12] each found that 66%, 63.1%, 68.8%, 69.5% and 73% of the females in their study were married respectively. Chavan et al. in Chandigarh reported contradictory findings, which found that 57.4% of his study population were unmarried [13].

Amongst all married women, seven (7.69%) women were divorced or separated from their husbands, and five (5.49%) women were widowed at the time of committing suicide, which was slightly similar to the findings of Prajapati et al. [11] and Khan et al. [12] who reported that 5.07% and 3% of their study population was widowed/divorced respectively. Pradhan et al. [7] contradict our findings when they found that the widowed population of their study was slightly higher (11%). These findings can be explained by the fact that; since the present study was conducted in a metropolitan city, where it can be expected the incidence of divorce would be higher.

In the present study, 36 (39.56%) of the married ladies committed suicide within seven years of marriage, followed by those married for 7-10 and 10-20 years accounting for 23 (25.27%) cases in each group. Such similarity was reported by Chakraborty and Rajan [14] who reported that 38.7% of married females committed suicide within seven years of marriage. Whereas, Srivastava and Arora reported that the majority of deaths happened within three years of marriage (60.01%) [15], and Verma and Misar reported that 45% of deaths occurred within one year of marriage [16]. Marriage is said to provide social support, but in our study, we find that the decedents didn’t find it in their marriages.

In the present study, the maximum numbers of victims committing suicide were in the age group of 26-32 years accounting for 34 (37.36%) cases, which coincides with the findings of Chakraborty and Rajan [14], who found that 40.3% of suicidal deaths were in the 3rd decade. Choudhary R [17] in her study also found the majority of females (88%) to be in between 18-35 years. However, in their study, Srivastava and Arora found a higher incidence in the age group 18-25 years [15], and Verma and Misar found the highest incidence in the age group of 21-24 years (43.85%) [16]. In the present study, this was followed by the age group of 18-25 years and 33-39 years accounting for 21 (23.07%) cases and 16 (17.58%) cases respectively, which is consistent with the findings of Prajapati et al. [11], Zine et al. [18] and Sahu et al. [19]. Only 10 (10.98%) cases were observed in the age group 40-49 years. The least number of cases were observed in the age group 50-60 years accounting for nine (9.89%) cases. This may be explained by the fact that being an urban setup, 26-32 years is the age most females get married or are in their early years of marriage. Being younger they are unable to cope with the various challenges of married life and choose suicide as an easy option to escape their predicament, challenges being dowry harassment or domestic abuse. Clearly, as the woman matures in age, she gets proficient in handling such matters, as shown by decreasing numbers in the next age groups.

As seen in Table 2, the majority (90%) of the married females committing suicide were literate, amongst which, 27 (29.67%) completed their middle schooling which coincides with other authors [8,9,11,14]. Dandona et al. [8] also reported that the suicidal death rate was higher among women who completed class 6. Prajapati et al. [11] found that 89.1% of their victims were literate and 30.44% had completed middle school. Chakraborty and Rajan [14] reported that 62.9% were literate and 29.8 % completed middle school. Contradicting findings were seen by Srivastava and Arora [15] who reported that the majority of their victims completed primary school (37.76%) followed by being illiterate (25.87%). In the present study, illiterate women accounted for nine (9.89%) cases, which contradicted with findings of Chakraborty and Rajan [14], who found that 37.1 % were illiterate. This finding can be explained by the fact that, since the present study was conducted in a metro city, there would be easy access to educational resources present, but could not complete more than their basic education.

Table 1 also shows that most victims were housewives accounting for 73 (80.21%) cases which is consistent with other authors [8,9,11,12,14,15,17-19]. Dandona et al. [8] reported that more than 50% of suicidal deaths were by housewives. Prajapati et al. [11] reported 71.74% of the victims as housewives and Chakraborty and Rajan [14] found that to be 71.8%. This shows us that the majority of the victims in the present study were dependent on their husbands or in-laws for financial support. Lack of financial independence makes these women reliant on their husbands or in-laws, stripping them of the ability to escape abusive marriages.

In our study, we also found that the most vulnerable socio-economic class for suicidal deaths in women is the socio-economic status-III (lower-middle class), accounting for 73 (80.22%) cases consistent with authors [7,15,16]. However, Zine et al. [18] found that the majority of their victims belonged to socio-economic status-IV (upper-lower class). Prajapati et al. [11] and Chakraborty and Rajan [14] showed that the majority of their victims belonged to socio-economic status- II (upper-middle class).

While analysing the cause of death, we found that the maximum number of decedents chose to commit suicide by hanging which accounted for 76 (83.51%) cases, was followed by consumption of an unknown poison which accounted for eight (8.79%) cases, which coincided with the findings of authors [7-9,15,17,19]. The findings of Prajapati et al. [11], Chakraborty and Rajan [14], and Sharma et al. [20] contradict our findings where they found that consumption of poison was the preferred method of committing suicide. Among the cases of poisoning, we found that the most common poison used was Organophosphate poison which was also reported by Chakraborty and Rajan [14]. In the present study, it was found that only four (4.35%) women chose to burn themselves to commit suicide which distinctly contradicts the findings of Verma and Misar [16], Sharma et al. [20], and Pawat et al. [21] studies in which the majority of females preferred self-immolation to end their lives. Whereas Khan et al. found that the most common method of committing suicide was by jumping into a river or lake [12].

Table 5 shows the most common cause for suicide in married females was physical and mental torture for dowry or domestic abuse which accounted for 21 (23.07%) cases. This was consistent with the study done by other authors [14,15,18]. According to Pradhan et al. [7] and Ramesh et al. [9] studies, the most common reason for committing suicide was marital disharmony, whereas Dandona et al. [8] reported that family problems were the most common reason reported for committing suicide. The next common indication in the present study for committing suicide in our study was anxiety as a result of ill health which accounted for 18 (19.78%) cases, similar to Prajapati et al. [11] who reported that 21.9% of suicides in their study was due to ill-health. Psychiatric disease or depression was the reason for committing suicide in 14 (15.38%) cases. In less than 10% of cases each, the causes were given as financial difficulties, grief due to the loss of a family member or ill health, trivial fights with the husband, failure of the marriage, and some form of sexual harassment in the decreasing order. In contrast, Choudhary R reported that for 46% of females committing suicide, the reason was anxiety and/or depression followed closely by domestic conflicts and violence (38.46%), as that study was done during the COVID lockdown period and during that time there was a lot of financial uncertainty and increased cases of domestic violence [17].

Bangalore is a metropolitan city in India, with multiple Tertiary care hospitals and Medical colleges within its limits. The present study was conducted at Kempegowda Institute of Medical Sciences, which caters to the southwest region of the city limiting the number of cases being brought to our centre for post-mortem, as a result of which the study sample was only 91 cases.

## Conclusions

This study shows us that, married women between 26-32 years of life who are homemakers and belong to the lower middle class of society are more at risk of committing suicide. This risk increases if there is abuse at the home front for dowry or abuse from her husband. Even though India has stringent laws against dowry and domestic abuse, the rate of suicide among married women is still alarming. This can be explained by the lack of support from the justice system, the ambiguous language of the law, the patriarchal cultural values, and the lack of economic opportunities for women. A marriage fails to provide protection to women in India from committing suicide in the form of a lack of spousal support. Dowry-related abuse or domestic abuse continues to be a driving force compelling married women to consider suicide as the best way to escape the situation. Despite the inroads made in female literacy, the expectations of women after marriage have not changed. The entire burden of managing the domestic chores falls on her. In spite of being educated, there is a presumption that she should sacrifice her interests and herself for her family, which is eroding her sense of self. Similarly, there is a lack of women empowerment and awareness of women-friendly laws at the grassroots level which leads to the rate of suicide amongst married women to be high.
